# Of Inflammation and the Therapeutical Application of Poultices and Fomentations

**Published:** 1857-02

**Authors:** H. P. Strong

**Affiliations:** Beloit, Wisconsin


ART. II.— *Of Inflammation and the Therapeutical Application of Poultices and Fomentations.* An Essay written for the Rock County Medical Association, by H. P. Strong, M. D., of Beloit, Wisconsin.

Poultices are become such a time-honored "institution," that he who would assail their application or doubt their efficacy, must indeed be presumptuous. They are considered to be the *sine qua non* for external application in all inflammations, whether sthenic, asthenic, acute, sub-acute or chronic; inaction, or passive congestions, in short, nearly all diseased action in the muscular, nervous, and cellular tissues are treated to a poultice. By some, cataplasms are prescribed for all varieties of ulcers, abscess, fistulas and sinuses indiscriminately. Every diseased part that looks as if it *might* have a pyogenic membrane is supposed to be materially benefited by the "softening process."

We, like Abernethy, like to cut the matter short and say: "A poultice, madam."

There are two things that injure our profession, and sometimes restrict the good we might do: The first is, our tendency to "hobbies," or favoritism; and the second, to ascribe more to therapeutical agents and less to what Cullen calls the *vis medicatrix naturæ*, than we should. Let us try to avoid these, and not do harm by carrying our favorite remedies too far, or by perplexing Dame Nature beyond her endurance.

The first thing to be considered is: Why do we resort to poultices and fomentations as medical agents? Because we have some local morbid action to subdue, either irritation, congestion, or inflammation, with their physiological and chemical tendency adverse to a healthy action of the animal economy.

Our next inquiry should be of the *rationale* of their application, or the *modus operandi* in effecting the desired result.

Before the advent of the immortal Harvey, while the circulation of the blood was unknown, and while the humoral pathology was so much in vogue, physicians, (according to Burns,) believing in the existence and influence of different humors and spirits, believed in the possibility of depositions and congestions of the blood, the bile, or lymph: and acknowledged these as the cause of inflammation. Their anatomists taught them that the liver was the centre of the vascular system, from which the blood went forth by day to the extremities and returned again at night. If then any peccant matter irritated the liver, the blood was sent out more forcibly. If any part of the body was weakened, irritated by heat or other agents, a swelling was produced by the flow of humors to this place, the peculiar nature of which was supposed to depend upon the kind of humor.

An idea was entertained that the blood and humors might slowly stagnate in a part from want of expulsive power, and this constituted a congestion.

These theories being incompatible with the locale of the circulation of the blood, we next have the theory of obstruction. Boerhaave, in his aphorisms, inculcated that inflammation was caused by an obstruction to the free circulation of the blood in the minute vessels, and this obstruction, he supposed, might be caused by heat, diarrhœa, &c., or whatever could dissipate the thinner parts of the blood, and produce viscidity of that fluid. He supposed that when such causes did not exist that the larger globules of the blood passed into the small vessels and thus plugged them up. He also advocated the acrimonious state of the fluids, which rendered resolution impossible and gangrene likely to follow. This local error is, doubtless, partially true.

From these incorrect theories, we have advanced to other, and more plausible ones of later days, and which, to our comprehension, approach much nearer the truth. Yet the fact that there are at present different, and partially contradictory theories, each sustained by great ability, is evidence to our minds that it is somewhat in obscurity even now. If the force by which the capillary circulation is sustained, was certainly demonstrated, we would have less trouble in arriving at truth; but as it is we have to adopt that course of reasoning which appears to be most conclusive.

Cullen attributed the proximate cause of inflammation to a "spasm of the extreme arteries supporting an increased action in the course of them."

Hunter considered the act of inflammation to be "an increased action of the vessels, which, at first, consists simply in an increase or distension beyond their natural size," and this increase seemed to "depend upon a diminution of the muscular power of the vessels." Owing to this increased action and dilatation, a greater quantity of blood circulated in the part.

Hastings thought Hunter's inferences would have been different had he viewed the inflamed vessels through the microscope, "for he would then have seen the blood moving, and found, that instead of its passage being quickened in the inflamed vessels, it is uniformly rendered slower in proportion to the degree of inflammation, and in the most inflamed parts stand still altogether."

Vacca, an Italian physician, in a small treatise, in 1765, was the first to put forth an opposing theory. He was seconded by Hastings, Wilson Philip, Thomson and others, who more at length contended that inflammation consists in the *debility* of the capillaries, followed by an increased action of the larger arteries, and is terminated by resolution, when the capillaries are so far excited, and the larger arteries so far weakened by the preternatural action of the latter, that the power of the capillaries is again in due proportion to the *vis a tergo*.

Thomson, from a series of experiments with the microscope, says: "It may be logically inferred, that inflammation is a weakened action of the capillaries, by which the equilibrium between the larger and smaller vessels is destroyed, and the latter become distended."

In modern times the vague but convenient expression "increased action of the vessels," has been very generally used as an adequate explanation of the proximate cause of inflammation. The doctrine is thought to derive support from a consideration of the exciting causes of the affection, which, being in general of an irritating nature, must, when applied to any living or sensible parts, occasion such increased action of the vessels; while the method of cure also leads to confirm the opinion. Whatever may be the facts elicited by the use of the microscope in regard to the dilatation of the capillaries, and apparent decrease of motion of fluid through them, there is no regard, or at least no conclusive evidence, showing whether the force by which the capillary circulation is carried on is at fault, or whether it be the increased action or excitement of the larger arteries leading to the inflamed part.

One great argument, and I might say almost *conclusive* argument against the supposition that the capillaries are in a state of debility, is, that the most effectual treatment of common inflammation consists in means which are generally of a debilitating nature, as the ordinary antiphlogistic remedies, bleeding, purging, antimony, application of cold, &c. Then we have still farther evidence of increased action by the recollection of the local augmentation of temperature, the throbbing, and the instantaneous return of the red color, after the discontinuance of any pressure by which the redness has been momentarily removed upon some point of the inflamed surface.

Microscopical experiments seem to show that in the capillaries of the part which is directly the seat of inflammation, there is a retardation and sometimes even a stagnation of the circulation. But this is not all: it is further manifested that the vessels are dilated, the blood altered, and that these phenomena are followed by an increased action of the larger arteries leading to the part affected.

Chelius, in speaking of inflammation, says: "The pain depends on the increased activity of the nerves, and this, again, produces the succeeding influx of the blood, and the vital expansion of the vessels; afterward the pain is increased by the decided expansion and tension which the part suffers. * * * * The redness, heat, and swelling, depend on the increased action of the nerves and capillary vessels, and are in immediate relation with the richness of their ramifications. Hence the various degrees of redness, heat, and swelling, according to the degree of inflammation, and the organs therewith affected. At the onset of the inflammation, the swelling always depends on an increase of blood. * * * * Where the most delicate branches of the capillary-vascular system anastomose to form the transition into the veins, several capillary vessels always open together into one single vein. By this disposition of the capillary-vascular system there is already in the healthy state a slower motion of the blood, which is in close relation to the functions of the capillary-vascular system. If, then, in inflammation there be an additional influx of blood, there must arise with the increased activity of the capillary-vascular system, and vital expansion, an accumulation of it (the blood) as the veins are not in a condition to take up and carry away with equal readiness, the blood which is brought to them in excess. The capillary vessels become, therefore, expanded as if filled by artificial injection, and even distinct in those parts where we assume that in the natural state vessels carrying only the uncolored part of the blood exist."

Believing, with Hunter, that inflammation consists in increased action of the vessels, at least in all acute sthenic inflammation, we pass to the resutls of inflammation, which are resolution, exudation, suppuration, ulceration and mortification.

In resolution the appearances of inflammation subside nearly in the same order as they were developed in the beginning, and the diseased part reverts to its natural condition again. Bennett has well described it as follows: "Resolution, or absorption of the exudation, may occur in various ways, and follow any of the transformations of the exudation except the one which converts it into permanent tissue. The early phenomena first disappear; the capillaries recover their contractility; the attraction between the blood and the parenchyma ceases: and the blood within the vessels begins to oscillate, and at length flows in a continuous stream. Secondly, the essential phenomena disappear, no further exudation takes place, and that already poured out is absorbed."

Exudation consists in the outpouring of a larger quantity of serous fluid than the capillary vessels ordinarily exhale into the cellular tissue, into the parenchyma of organs, or into the cavity of the body, and this fluid consists of serum and coagulable lymph, differing somewhat in its composition.

If inflammation passes beyond the preceding conditions, we then have suppuration, which consists of the secretion of pus through the walls of the capillary vessels, not, however, immediately as such, but through the changes which the coagulated fibrin of the inflammatory exudation undergoes, which mixes with the serum after being converted into pus-globules. Pus is formed principally from the albumen and fibrin of the blood, but somewhat from all its components with the exception of the coloring matter. Some have supposed it a vital, while others have considered it it a chemical process. The former believe that pus is formed and secreted within the vessels of inflamed organs by the peculiar activity of the former. The latter that pus is produced externally to the vessels of the inflamed organs, either in the solid parts, or in the effused fluids undergoing a change similar to that of fermentation or putrefaction.

Chelius is of the opinion that the process of suppuration is a true secretion, and the vital condition of the organs influences it as well as all other secretions, and that there is usually no destruction of tissues connected with suppuration. He thinks that the remnants of destroyed cellular tissue often found in pus, depend on accidental circumstances.

Hunter's theory, which is generally adopted as the modern one, is, that the matter is separated from the blood by the secreting power of the vessels of the inflamed part, which acquire a new mode of action.

When the interior of an abscess is examined, the cavity which contained the matter is observed to be lined with a smooth, membrane-like substance, which is of a whitish ash-color, and has a strong resemblance to coagulating lymph. This membrane-like investment has been termed the sac or cyst of the abscess, and more latterly, the pyogenic membrane, which is supposed also to have the power of secreting pus.

By ulceration and mortification we understand that a diseased part has passed beyond résolution to dissolution and death of the living solid.

Having thus considered inflammation and its results, we now turn more directly to the subject and inquire what is the effect of poultices and fomentations when applied to an inflamed part. They seem to sooth the sensitive operation of the nerves, and by keeping the cuticle moist and warm, exert a powerful influence in promoting suppuration. This is a fact that will be admitted by every experienced practitioner. My observation concerning the use of these remedies has inclined me to the opinion that there are some who yet believe in the humoral pathology, and in the doctrine of the surgeons of old, that the theory of suppuration is the dissolution of the living solids of an animal body into pus, and that this fluid has the power to continue the dissolution, so long as the doomed portion of the animal tissue remains. Upon the ancient principle that the solids are dissolved into pus, was founded the practice of bringing all indurated parts to suppuration, and so we have yet the practice of "softening indurated parts," and "encouraging the separation of the slough or core," in suppurating surfaces or cavities.

We have seen from the nature of inflammation, that we must assist Nature by antiphlogistic remedies—the application of cold, &c., in her first effort at restoration by resolution. The untimely adopted course of poulticing in the earlier stage only baffles Nature and prolongs the difficulty to her second effort at restoration by suppuration. We have our indications from the process of resolution, which consists in the capillaries recovering their contractility, and in the activity of the absorbents. Too often are the remedies here used contra-indicated, and resolution only takes place through the power of Nature in overcoming both the morbid action and the suppurating effect of poultices.

Cooper says that warm emollient applications of all kinds have a powerful influence in promoting suppuration, and the use of such remedies, while the resolution of inflammation is practicable, must be highly censurable.

Hunter says: "When poultices and fomentations are applied to inflamed parts, in which we wish to avoid suppuration, reason and principle will not justify the practice."

When we are certain that resolution will not take place, which will be shown by the increase of all the different symptoms, and particularly when the parts become soft, attended by a more violent throbbing pain, then we may conclude that suppuration will follow. *This* will be the proper time for poultices and fomentations, and I may say that this is nearly the *only* time when they should be used. They are the chief local means both of promoting suppuration and diminishing pain, violent throbbing, &c., which always precede this termination of phlegmonous inflammation. They act by enlarging the suppurating space whereby approach to the surface is effected, and by hastening the suppurative action. Here they are the *sine qua non* in our treatment; and they are so good that before aware of it, we are doing harm by the protracted application. From my own experience, and from observation, I am satisfied that much more harm follows their use at too late, than at too early a period.

Hear what Prof. Miller says: "But poulticing may be, and often is, over done. If continued after resolution of both vascular action and its structural change, harm is done by over-relaxation of texture, maintenance of congestion, and consequent prolongation of redundant discharge. True, inflammation is necessary to the first establishment of purulent secretion, but congestion, either active or passive, is equal to its maintenance; and a part constantly sodden by a hot and moist poultice cannot be otherwise than congested. There is no doubt that many open abscesses, and many suppurating wounds, are kept from healing, and an exhausting or hectic effect produced on the system, by an undue continuance of poulticing. The first two or three days after opening, usually suffice for subsidence of the major part of the inflammatory process, and then the poultice is to be superseded by the simple water dressings, applied tepid; not with an antiphlogistic view, but merely protraction, soothing, and abstergent."

The truth of these remarks were felt, and my attention more particularly called to this subject, by having presented for treatment, a case of a large sloughing suppurating ulcer on the face, which, upon inquiry, I learned began as a simple suppurating sore or boil. This had been poulticed for weeks successively, by order of the medical attendant, and no doubt *would* have been the order for time indefinite, had he remained under his charge. I need hardly say that cerate dressings and a stimulant wash effected a cure.

Poultices and fomentations too long applied, not only produce a redundant discharge, but favor the production of the pyogenic membrane of pus secreting functions, and thereby convert the abscess or surface into a chronic suppurating sore, and if co tinued long enough, into a sloughing sore: and when this is effected, a continuance of such applications may prolong the case until hectic fever declares itself.

Perhaps I have noticed this abuse, (and I claim no exemption myself formerly) in the treatment of common whitlow. We have all, doubtless, seen the stiff joints, contracted tendons, amputated fingers, and been more or less perplexed from these painful and troublesome things. Latterly I have thought this somewhat needless, and have ascribed much of the difficulty to a desperate persistence in the application of poultices, which sometimes, yes, *often*, produce that atonic form of erysipelatous inflammation which is so hard to bring to resolution or granulation, and which leaves such unpleasant effects. The true method in this difficulty is, I believe, to open thoroughly by a deep incision, and poultice until suppuration is established, and then inject a ten grain solution of nit. silver or similar preparation, and instead of having a suppuration of several weeks duration, and destruction of parts, you will have granulation and healing in a few days.

Anthrax is another of those forms of inflammation which are often maltreated in this way, inducing more sloughing and suppuration than necessary. This, with other manifestations of inflammation, are equally benefited by the course hinted. At all events, if we do not interfere to baffle the salutary operations of Nature in the way heretofore mentioned, we will have less cause to feel the truth of Miller's assertion, that "*nimia diligentia* is but sorry surgery."

